# Quality of life and work status in patients treated for neurogenic thoracic outlet syndrome via the microsurgical supraclavicular approach – an observational study

**DOI:** 10.1016/j.bas.2026.106266

**Published:** 2026-08-10

**Authors:** J. Alsolivany, J. Reinsch, T.A. Sargut, S. Baruchi, N.F. Dengler

**Affiliations:** a Department of Neurosurgery, Charité Universitätsmedizin Berlin, Corporate Member of Freie Universität Berlin, Humboldt-Universität zu Berlin and Berlin Institute of Health, Charitéplatz 1, Berlin, 10117, Germany; b Faculty of Health Sciences Brandenburg, Medical School Brandenburg Theodor Fontane, Fehrbelliner Straße 38, Neuruppin, 16816, Germany; c Department of Neurosurgery, HELIOS Hospital Bad Saarow, Pieskower Straße 33, Bad Saarow, 15526, Germany

**Keywords:** Neurogenic thoracic outlet syndrome, Quality of life, Return to work, Outcome, Supraclavicular approach

## Abstract

**Objective:**

Neurogenic thoracic outlet syndrome (nTOS) is a diagnostic and therapeutic challenge. Recently, consensus statements were developed within the neurosurgical community to improve diagnosis, classification and therapy. However, a standardized outcome assessment according to a validated Quality of life (QoL) questionnaire and has never been performed in patients surgically treated according to the consented recommendations.

**Objective:**

To provide systematic information about QoL based on a validated questionnaire and work status in patients surgically treated for nTOS via the supraclavicular approach without routine first rib resection.

**Methods:**

Patients surgically treated for nTOS between January 1, 2015, and June 30, 2023 were included. A systematic outcome evaluation using the EQ-5D-5L and a structured interview was performed.

**Results:**

The final analysis included 18 patients (median age 39.5 years [45-34], 71% female), of which 94.4% showed a benefit from surgery after a median follow-up (FU) of 1.7 years (IQR 0,7-3). Pain scores on the numerical rating scale (NRS from 0 to 10) significantly improved from 8 (IQR 9-8) to 4 (IQR 5-4) at FU (p < 0.001). The individual health-related QoL on a scale from 0 to 100 was rated with a median of 65 (IQR 50-75) before surgery and 85 (IQR 80-95) at FU (p < 0.001). Five dimensions (mobility/self-care/usual activities/pain/anxiety) were rated on scales from 1 (no problems) to 5 (inability to perform tasks) resulting in median values of 3/3/4/3/3 before surgery and of 2/2/2/2/1 at FU. Patient absence from work due to sick leave decreased from 44% to 18% at FU (p = 0.02).

**Conclusion:**

Consensus-based patient management in nTOS led to a significant benefit in terms of pain relief and individual health-related QoL. After surgery, return to work increased significantly.

## Highlights

• Microsurgical supraclavicular decompression for neurogenic thoracic outlet syndrome (nTOS) significantly improved pain, neurological symptoms, and quality of life.
• Health-related quality of life measured with the EQ-5D-5L improved significantly after surgery, with median scores increasing from 65 preoperatively to 85 at follow-up.
• Significant postoperative improvements were observed across all EQ-5D-5L dimensions, including mobility, self-care, usual activities, pain, and anxiety.
• The majority of patients (94.4%) reported clinical improvement following surgery without routine first rib resection.
• Surgical treatment significantly reduced work absenteeism and facilitated return to work in patients with nTOS.

## 1 Introduction

Neurogenic thoracic outlet syndrome (nTOS), the compression of nerval structures at the thoracic outlet, is the most common form of presentation among all forms of thoracic outlet syndromes (Thoracic Outlet Syndrome, 2019; Laulan et al., 2011). Persistent compression of neurogenic structures within the thoracic outlet may lead to a myriad of neurological symptoms impacting individual daily activities, quality of life (QoL), and professional pursuits (Dahlstrom and Olinger, 2012; Illig et al., 2021). The incidence is estimated at 2-3 cases per 100,000 persons/year with a predominance in females (Illig et al., 2021).

Various concepts of diagnosis, classification and therapy are applied to this patient group as management may be multidisciplinary (Dengler et al., 2022a, 2022b; Ferrante, 2012). Recently, a consensus among neurosurgeons on patient management was reached (Dengler et al., 2022a, 2022b), recommending the microsurgical supraclavicular approach without routine first rib resection by most neurosurgeons (Brooke and Freischlag, 2010; Rochkind et al., 2023). The advantageous features of this approach include precise intra-operative diagnosis, secure identification and release of all abnormal anatomical pathologies and comprehensive visualization of the entire plexus without unnecessary morbidity (Scali et al., 2010; Ferrante and Ferrante; Huang and Zager, 2004).

For brachial plexus lesions, a validated QoL outcome instrument such as the EQ-5D-5L is recommended (Feng et al., 2021). However, in nTOS, to date, systematic QoL data based on a validated questionnaire and data on patients' work status are lacking. Therefore, the presented study is the first to assess QoL, based on the validated EQ-5D-5L questionnaire, and return to work for nTOS patients treated by a microsurgical decompression via the supraclavicular approach without routine first rib resection.

## 2 Methods

### 2.1 Study design and acquisition of data

Ethical approval of the study was granted by local authorities (01/034/21). The patients consent to the procedure and the publication of their images. The institutional database of patients with peripheral nerve disease was screened for nTOS cases surgically treated by a single surgeon (NFD) between January 1, 2015 and June 30, 2023. Inclusion criteria were the diagnosis of nTOS according to the following nTOS subclassification that was recently published by the Peripheral Nerve Section of the European Association of Neurosurgical Societies (EANS) (Dengler et al., 2022b):

- nTOS 1 - Hypotrophic nTOS: This subgroup comprises patients presenting with muscle weakness, hypotrophy, or atrophy primarily affecting the upper limbs, usually in a distal pattern.
- nTOS 2 - Irritative nTOS with anatomical anomaly: Patients in this subgroup exhibit pain or sensory symptoms without motor deficits.
- nTOS 3 - Irritative nTOS without anatomical anomaly: This is a diverse subgroup encompassing patients without motor involvement who experience pain or sensory symptoms.

Other inclusion criteria were age ≥ 18 years, failed conservative management by physiotherapy and/or pain medication, surgery via the microsurgical approach, and exclusion of a cervical pathology by cervical MRI or a peripheral pathology (carpal or cubital tunnel syndrome) by electrophysiology. The HRQoL, was assessed using the EQ-5D-5L questionnaire that was sent to all patients. The EQ-5D-5L consists of two sections. Problems in five dimensions of daily living (mobility/self-care/usual activities/pain/anxiety) are quantified on a scale from 1 (no problems) to 5 (not able to perform tasks) within the first section. The individual HRQoL is quantified on a visual analogue scale (VAS) ranging from 0 to 100 within the second section of the EQ-5D-5L (Feng et al., 2021). The following variables were assessed at routine follow-up examinations or by a structured telephone interview: age, sex, duration of symptoms before surgery, intraoperative findings in terms of anatomic abnormalities, self-reported benefit from surgery, pain on the numeric rating scale (NRS, 0-10 points), time to follow-up, HRQoL at follow-up, and work status. Comorbidities and body mass index (BMI) were documented.

### 2.2 Treatment algorithm

**Fig. 1:**
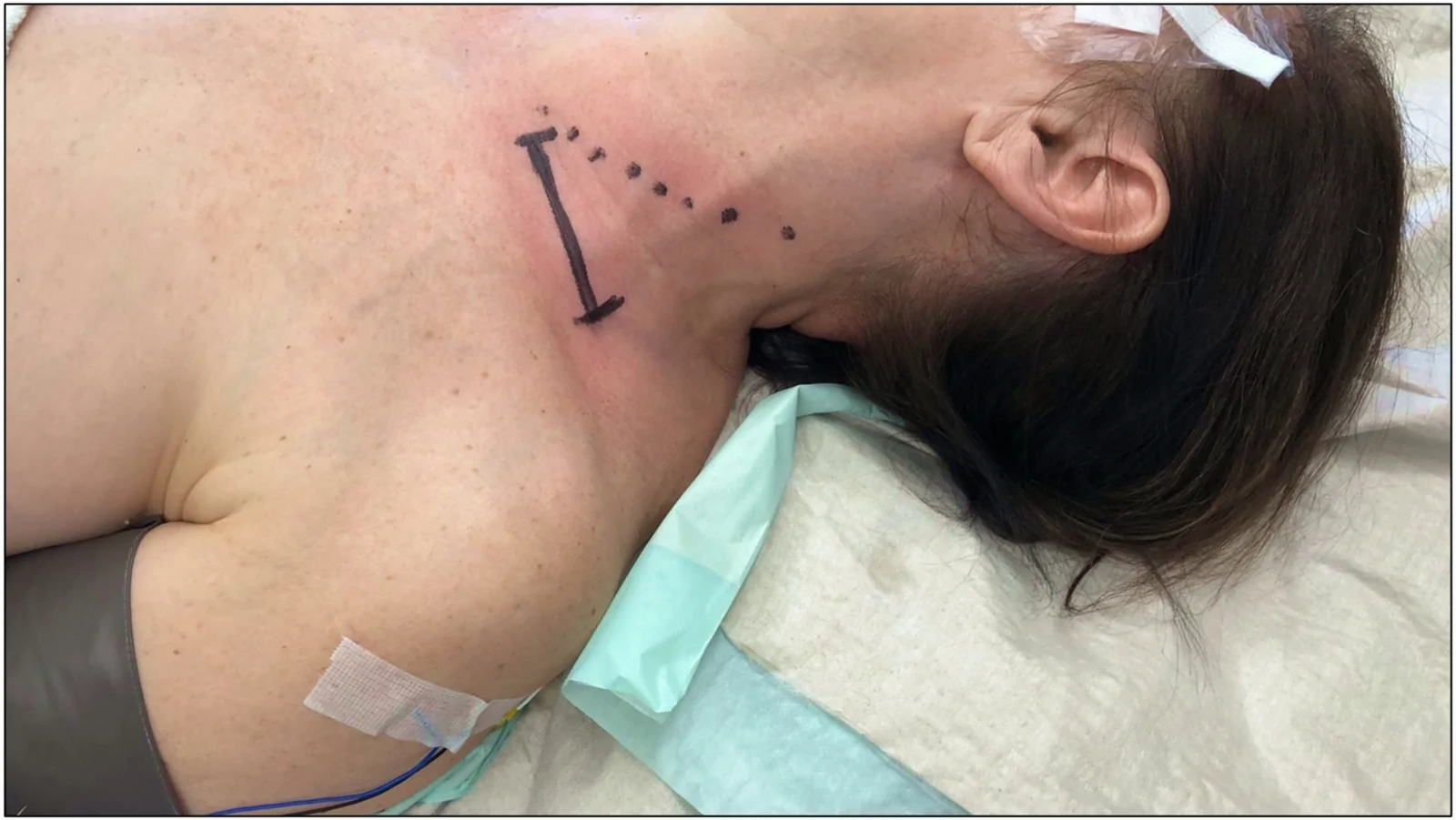
Intraoperative patient positioning. The arm of the affected side (here left arm) is extended and positioned on a side table. The head is turned against the contralateral side. The incision line (continuous) is approximately two fingers above the clavicle. The sternocleidomastoid muscle is marked by the dashed line.

Patients were evaluated in our outpatient clinic prior to surgery. All patients underwent an x-ray of the thoracic outlet, an MRI or ultrasound of the thoracic outlet and an MRI of the cervical spine, as recommended (Dengler et al., 2022a, 2022b). Other peripheral nerve pathologies (e.g., carpal or cubital tunnel, loge de Guyon syndrome) were ruled out by electrophysiological examination, or, if present, primarily addressed. In case of nTOS 2 and nTOS 3, patients underwent an initial conservative treatment phase with pain management and/or physiotherapy. In case of persisting symptoms surgery was offered according to recent consensus recommendations (Dengler et al., 2022b; Rochkind et al., 2023). Surgery was performed in a supine position as described elsewhere (Ferrante, 2012; Dengler et al., 2022b). In short, the arm of the affected side is extended and positioned on a side table. The head is turned towards the contralateral side (Fig. 1). Intraoperative electrophysiological monitoring with free-run electromyography EMG) of the M. opponens pollicis, M. flexor digiti minimi, M. deltoideus and *M. biceps* brachii as well as a monopolar stimulation probe is established. A 3 cm incision approximately two fingers above the clavicle is marked. Preparation based on the important landmarks including the sternocleidomastoid muscle, the supraclavicular fat pad, the omohyoid muscle, the phrenic nerve, and the anterior scalene muscle is performed until the brachial plexus is visualized. Further preparation is performed under microscopic view using a retractor. The superior, medial and inferior trunks are visualized. A three-dimensional decompression is performed by partial anterior scalenectomy, transection of fibrous bands, resection of bony anomalies and decompression of the subclavian artery. The first rib is visualized. Position maneuvers are performed to check for adequate decompression in provocative positions. The first rib is only resected in case where residual compression is detected. Free-run EMGs are constantly checked.

### 2.3 Statistical analysis

Statistical analysis was performed using SPSS software (Version 25.0. Armonk, NY: IBM Corp.). All values were expressed in numbers and % of patients or median and interquartile range (IQR) and were compared using the non-parametric Mann-Whitney U or Wilcoxon test for metric non-normally distributed variables and Chi (Laulan et al., 2011) test for ordinal or nominal scaled variables. Statistical significance was assumed at p-values of <0.05.

## 3 Results

### 3.1 Patient characteristics

Among 21 patients, 85.7% responded to the questionnaire, resulting in a total of 18 patients included into the final analysis. The median age was 39.5 years (IQR 45- 34). The majority of patients was female (71%). Comorbidities were present in 44% of patients. Hypertension was the most frequent comorbidity (present in 22% of patients), followed by diabetes mellitus (17%), hypothyroidism (17%), and obesity (11%).

Among the study participants, three patients had a history of smoking, while the majority (15 out of 18, 83%) had no smoking history. The follow-up assessment was conducted at a median of 1.7 years after the surgical intervention (IQR 0.7-3) (Table 1).

**Table 1:** Characteristics of the nTOS patient cohort.

Patient number	18
Median age in years (IQR)	39.5 (45-34)
**Sex** (n)	72% (13)
Female	28% (5)
Male	
Symptoms (n) Motor deficits Sensory deficits - Hypesthesia - Paresthesia - Pain (94%)	17% (3)
	100% (18)
	100% (18)
	56% (8)
	94% (17)
**Side of symptoms** (n)	17% (3)
Both	61% (11)
Right	39% (7)
Left	
Median duration of symptoms before surgery in months (IQR)	48 (96-24)
**Anatomical variations** (n) - Accessory cervical rib - Accessory muscle - Sibson fascia - Prolonged transverse C7 processus	28% (5)
	61% (11)
	11% (2)
	5% (1)
**nTOS-Classification** (n)	17% (3)
nTOS 1	83% (15)
nTOS 2	0% (0)
nTOS 3	
**Presence of comorbidities** (n)	44% (8)
Hypertension	22% (4)
Hypothyroidism	17% (3)
Diabetes mellitus, type II	17% (3)
Obesity	11% (2)
**Work status** (n)	67% (12)
Absent from work	17% (3)
Retired	
**Increased motion of upper extremities due to (n)** - Work - Physical activities	28% (5)
	22% (4)
**Preoperative pain medication** (n)	78% (14)
Yes	22% (4)
No	
**Smoking history** (n)	17% (3)
**BMI** (IQR)	23.4 (27.4-21.7)
Median	23.1 (23.9-21.1)
Female	27.7 (37.3-24.3)
Male	

Abbreviations**:** nTOS – neurogenic thoracic outlet syndrome, IQR – interquartile range, n – number of patients, BMI – body mass index.

A detailed illustration of the clinical features, nTOS classification, and outcome at follow-up in our nTOS patient cohort is further demonstrated in Table 2.

**Table 2:** Detailed illustration of clinical features, nTOS-Classification, and outcome at follow-up in our nTOS patient cohort.

Pat.-ID	Age	Gender	BMI	Length of symptoms (months)	Smoking	Pain Preop (NRS)	nTOS-class. (n1-3)	Work status preop	Age at surgery	Side	EQ-5D-5L Preop (VAS)	EQ-5D-5L Mob/SC/Act/Pain/Anx	Time of FU (months)	Pain at FU (NRS)	EQ-5D-5L at FU (VAS)	EQ-5D-5L Mob/SC/Act/Pain/Anx	Work Status at FU
1	21	F	26.8	20	No	8	n2	Absent	21	Left	80	1/4/4/3/2	9	4	95	1/2/2/2/1	Absent
2	31	F	27.9	23	No	7	n2	Absent	30	right	80	1/2/2/2/1	18	2	100	1/1/1/1/1	Working
3	31	F	19.5	23	No	8	n2	Working	31	right	70	4/4/4/4/3	15	3	70	2/3/3/3/1	Working
4	37	F	28	60	No	9	n2	Retired	37	right	60	4/3/4/4/4	7	6	85	2/2/3/2/2	Retired
5	67	F	22.2	18	No	6	n2	Absent	66	right	80	2/1/2/3/3	19	4	90	2/1/1/2/2	Working
6	21	F	22	43	No	8	n2	Absent	21	right	90	2/1/2/3/3	25	6	95	2/1/2/1/2	Absent
7	45	F	21.7	34	Yes	9	n1	Working	40	right	50	2/4/4/4/4	82	7	95	1/2/2/2/2	Working
8	35	F	18	42	No	8	n2	Absent	35	right	60	3/3/4/4/3	28	7	80	2/2/2/3/2	Working
9	44	F	23.9	60	No	8	n2	Retired	44	right	65	3/3/4/4/3	58	4	85	1/2/2/1/1	Retired
10	70	M	23.1	34	No	7	n1	Absent	70	left	65	2/2/3/4/1	57	8	65	2/2/3/4/1	Absent
11	41	M	24.3	67	No	8	n1	Retired	37	left	60	3/4/3/4/3	6	5	85	2/3/2/2/1	Retired
12	69	M	37.7	54	No	9	n2	Working	69	left	50	3/3/4/4/3	37	2	95	2/2/2/2/1	Retired
13	44	M	27.7	55	No	8	n2	Absent	54	right	40	3/3/3/4/4	12	8	70	2/2/2/2/2	Working
14	54	M	38.1	28	Yes	9	n2	Working	54	left	40	2/3/3/3/3	36	2	80	1/2/2/1/2	Working
15	40	F	19.4	40	Yes	10	n2	Absent	40	left	50	4/4/4/3/4	2	5	75	2/2/2/2/2	Absent
16	34	M	23.5	34	No	8	n2	Working	34	left	60	4/3/4/3/3	3	3	80	2/3/3/2/3	Working
17	39	M	21.1	28	No	9	n2	Working	42	right	65	4/3/3/4/3	2	4	80	2/2/3/2/2	Working
18	40	F	23.3	38	No	3	n2	Absent	40	right	50	3/4/4/2/4	6	3	80	1/1/2/2/2	Working

Abbreviations: Pat.-ID - Patients-Identification-number, BMI – Body Mass Index, Preop - preoperative, NRS – numeric rating scale for pain from 0 to 10, VAS - Visual-Analogue Scale for individual health related quality of life on a scale from 0 to 100, nTOS – neurogenic Thoracic outlet syndrome, class. – Classification, FU - follow-up, Mob – Mobility, SC – Self-Care, Act – Activity, Anx - Anxiety.

### 3.2 Surgical outcome

**Fig. 2:**
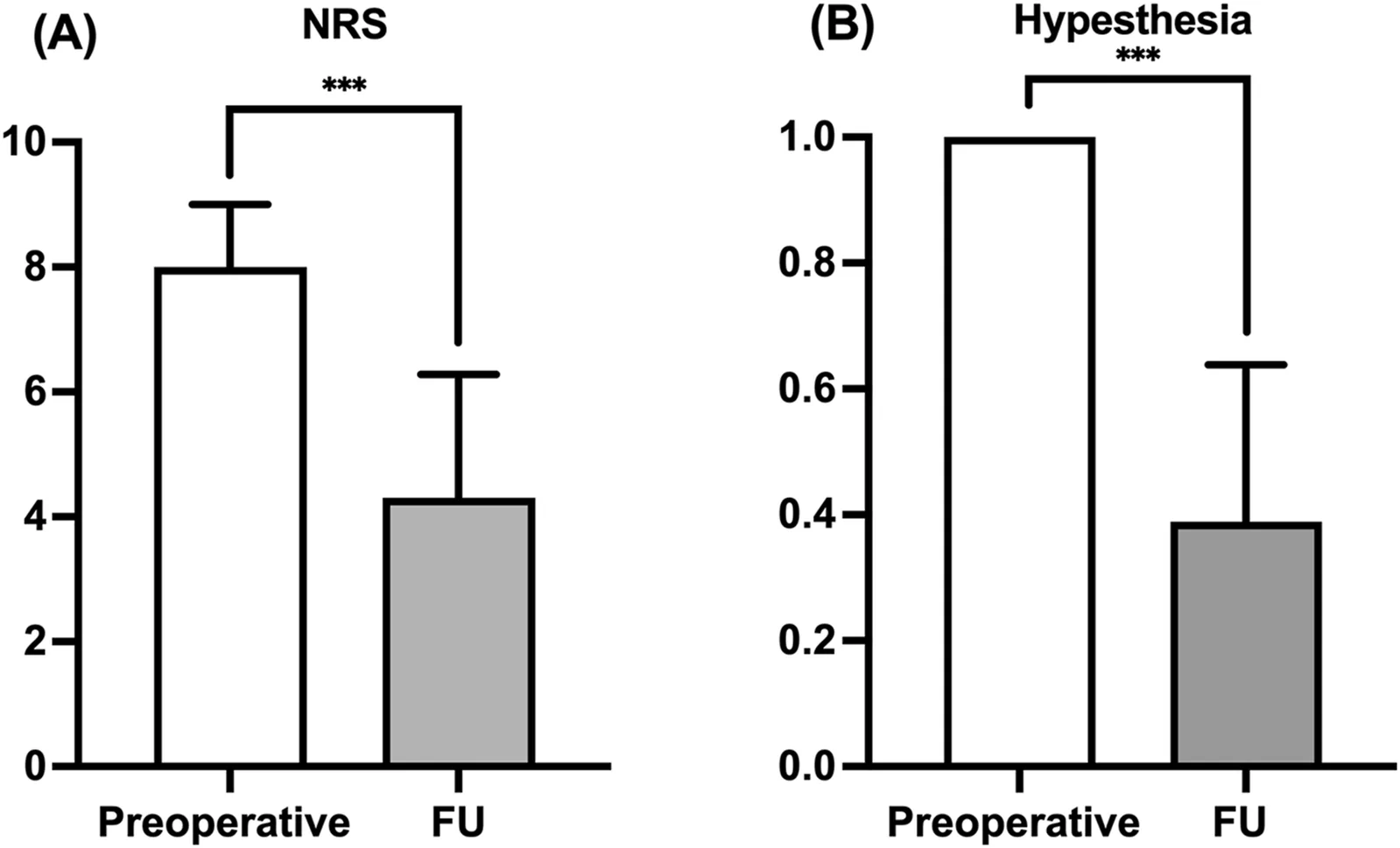
Surgical outcome with respect to symptom improvement. A) pre-and postoperative pain levels in patients with nTOS using the numerical ratings scale (NRS) from 0 to 10. B) Pre- and postoperative percentage of patients with hypesthesia. (***) stands for a significance level of p < 0.01.

In 94.4% of patients a clinical improvement was noted after surgery. Prior to surgery, the median NRS pain score was 8 (IQR 8-9) which significantly decreased at follow-up (FU), to a median pain score of 4 (IQR 3-6) (p < 0.001) indicating substantial benefit from the surgical procedure (Fig. 2 A).

The number of patients with hypesthesia decreased from 18 (86%) preoperatively to 7 (33%) at FU, (p < 0.01) (Fig. 2 B). The number of patients with motor deficits decreased from 3 (14%) to 1 (5%) at FU, (p < 0.01) respectively.

### 3.3 Quality of life

**Fig. 3:**
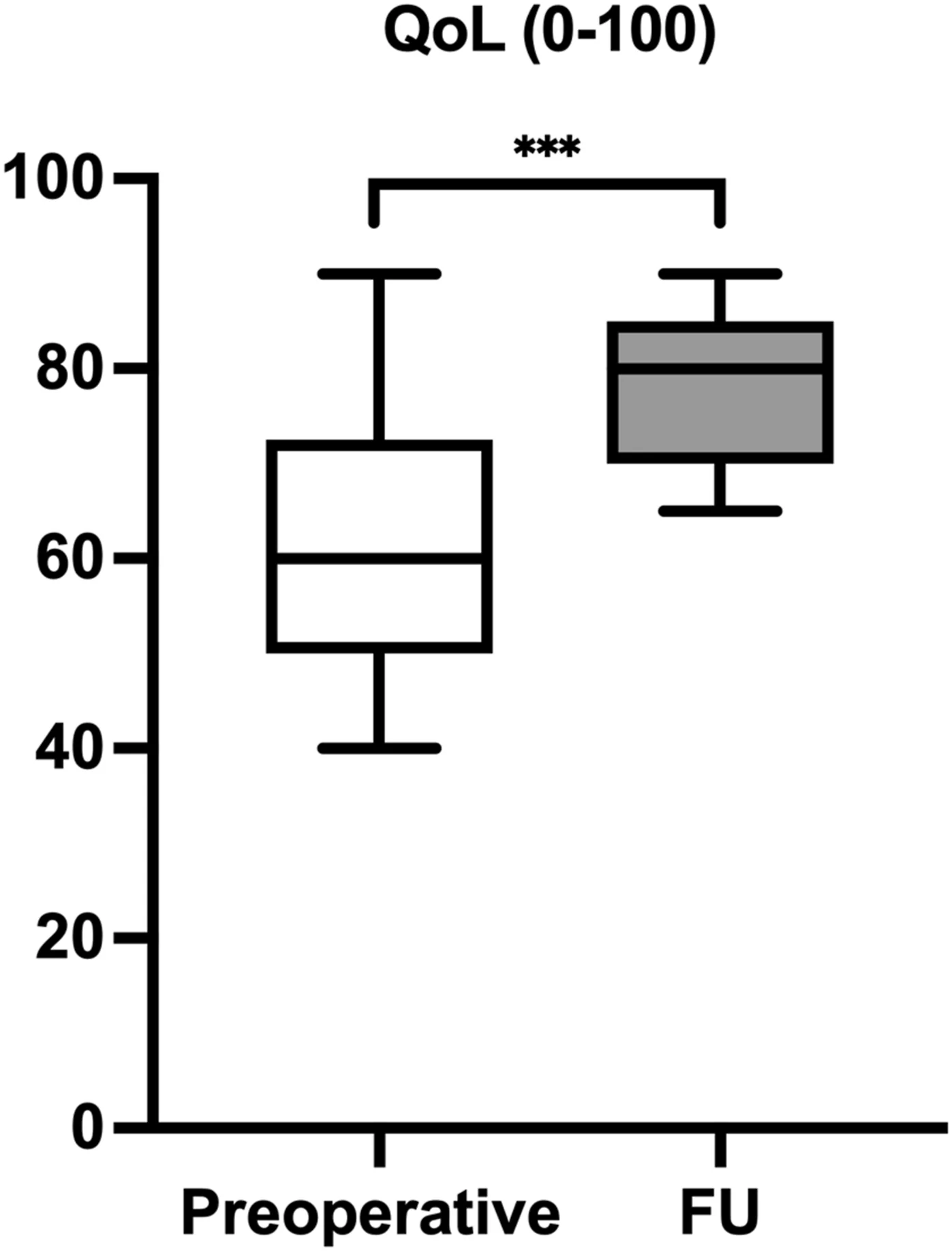
Quality of life (QoL) in patients surgically treated for nTOS. Comparison of preoperative QoL versus FU using a scale ranging from 0 to 100 presented by a boxplot showing the median as well as the lower and upper quartiles. (***) stands for a significance level of p < 0.01.

**Fig. 4:**
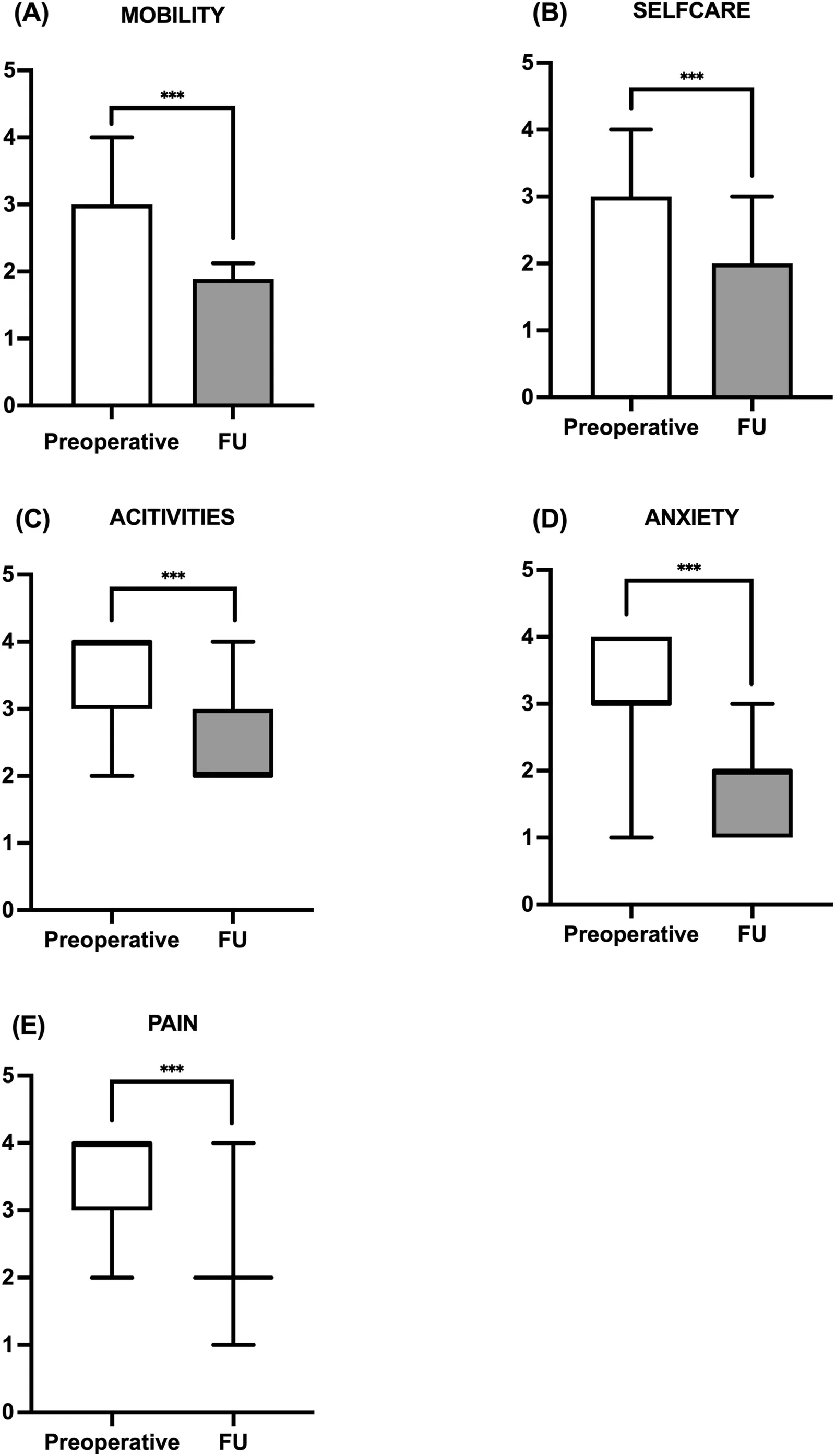
Quality of life in patients surgically treated for nTOS. Dimensions illustrated: A) Mobility, B) Self-care, C) Activities, D) Anxiety and E) Pain. (***) stands for a significance level of p < 0.01.

Before surgery, the median QoL score was 65 (IQR 42-80). At FU, a significant improvement to 85 (IQR 75-95) (p < 0.001) was noted, indicating a substantial enhancement in overall well-being and functional status following surgery (Fig. 3). On a scale from 1 (no problems) to 5 (inability to perform tasks) the various dimensions of patient well-being (mobility/self-care/activity/pain/and anxiety) were rated. Before surgery, median scores were 3/3/4/3/3 in the respective dimensions. At FU, all dimension ratings improved to median scores of 2/2/2/2/1, respectively (Fig. 4A–E).

### 3.4 Work status

Prior to the surgery, a considerable proportion of patients (68%) was absent from work. Among these, 41% were on sick leave, and 17% retired. At FU assessment, a notable reduction in work absenteeism was observed. The percentage of patients absent from work decreased to 44%, with 18% on sick leave and 23% retired (p = 0.02), resulting in an overall return to work of 14%

## 4 Discussion

### 4.1 Clinical efficacy of supraclavicular decompression in nTOS

In our study cohort, patients with nTOS treated according to recent consensus recommendations benefitted substantially from surgical decompression via the supraclavicular approach with significant improvement in pain, hypesthesia, and motor symptoms. Our data show that this approach was beneficial for improvement of QoL in patients with nTOS. This is not only true for the individually rated health related QoL, but also for the different dimensions in terms of activity, self-care, pain, and anxiety. Moreover, a significant positive impact of surgery on individual return to work at was observed.

### 4.2 Surgical strategy and anatomical considerations

Studies specifically addressing the surgical outcome of nTOS are limited and former TOS classifications systems are generally used heterogeneously (Dengler et al., 2022a; Rochkind et al., 2023). A recent consensus on classification reached by neurosurgeons now allows for more differentiated patient evaluation and categorization (Dengler et al., 2022b; Rochkind et al., 2023).

According to our data, most patients were classified as nTOS 2. A structured analysis of nTOS outcome reports after microsurgical decompression via the supraclavicular route without routine resection of the first rib is currently lacking given that most studies examine mixed TOS populations as well as transaxillary approaches and surgical strategies with routine first rib resection (Thompson, 2012; Peek et al., 2017; Nejim et al., 2017):

Alternative surgical strategies for nTOS include the transaxillary approach and infraclavicular or combined approaches, most commonly in combination with routine first rib resection (Thompson, 2012). While the transaxillary approach provides direct access to the first rib, visualization of the brachial plexus and associated anatomical variations is more limited compared with the supraclavicular microsurgical approach (Thompson, 2012; Peek et al., 2017). In contrast, the supraclavicular approach allows comprehensive exposure of the brachial plexus, identification of anatomical abnormalities, targeted neurolysis, and selective first rib resection only when persistent compression remains after soft tissue decompression. As comparative evidence between these surgical strategies remains limited and no randomized studies are available, superiority of one approach over another cannot currently be established. Surgical approach should therefore be individualized according to anatomical findings and surgeon expertise (Peek et al., 2017; Nejim et al., 2017).

In our cohort, first rib resection was performed only if compression was still present after resection of anatomic variabilities such as a cervical rib or a prominent C7 transverse process, and after the transection of fibrous bands, muscles or other soft tissues. This was the case in three patients that had a persistent compression of the brachial plexus after resection of fibrous bands and muscular decompression due to the individual anatomy of the topographic relation between first rib and brachial plexus. In our cohort, a benefit from surgery was detected in 94% of subjects. Our results indicate that a significant reduction in pain levels (NRS improvement from 8 to 4) was achieved post-surgery. No complications such as pneumo- or chylothorax occurred in our patient cohort. Our data has to be interpreted in the context of other reports on patients, which were not treated according to the recent consensus recommendations used in our series (Povlsen et al., 2014; Rochlin et al., 2013). For example, in the study by Rochlin and colleagues in patients treated with transaxillary scalenectomy and routine first rib resection symptom improvement was detected in only 72% of patients and pneuromothoraces occurred in about 22% of patients while EQ-5D-5L scores were not examined (Hinz et al., 2014). In a series published by Bosma and colleagues nTOS surgery failed to improve QoL to ranges of healthy people (Rochlin et al., 2013).

### 4.3 Quality of life and impact on mental health

Chronic pain is known to have a profound impact on the physiological and psychological well-being, often leading to increased levels of stress, reduced activity, anxiety, and depression (Wyns et al., 2023;Bosma et al., 2010). Outcome reporting for nTOS patients including factors associated with well-being in daily life is scarce (Peek et al., 2017).

The EQ-5D-5L, which was used in our analysis allows for comparisons with the general population as well as for cross-country comparisons due to previous validation studies (Wilson et al., 2024;Marten and Greiner, 2021). In our cohort, the individual health-related QOL prior to surgery ranged at a median of 65, which is remarkably below the German average population, for which it ranges between 73 and 92^25^. The significant increase to 85 observed after surgery showed that nTOS patients operated on using h microsurgery via the supraclavicular approach, can reestablish or even surpass normal QOL scores.

The substantial improvement in activity and mobility observed in our cohort after surgery may be interpreted as a direct result of decompression of the affected neural structures. This may enhance the ability to perform daily activities but also empower patients them to engage in physical activities previously avoided (Pesser et al., 2021; Ludwig et al., 2018).

Evidence on patients afflicted with chronic pain syndromes or peripheral nerve compression syndromes established a link between mental health and the postoperative recovery process (Alnahhal et al., 2023; Córdoba-Mosqueda et al., 2023; Beith et al., 2011). Anxiety decreased significantly after surgery in our cohort of nTOS patients. This is in line with the reduction of pain and the decrease and thus improvement of the sensorimotor deficits and reintegration into daily life. Nevertheless, these results should be discussed cautiously, as, to date, there is no other study addressing the issue of anxiety in patients with nTOS undergoing surgery.

Given that the average age of patients with nTOS ranges between 30 and 40 years, thre is relevant impact on the working demographic, making return to work a pivotal outcome parameter in this patient group (Laulan et al., 2011; Ludwig et al., 2018). A notable challenge associated with nTOS lies in the protracted duration required for diagnosing the condition. This may leads to severe limitations in daily activities and an incapacity to work during the prolonged diagnostic process preceding definitive treatment (Dengler et al., 2022b; Thompson, 2012). The predominant symptomatic manifestations of nTOS, characterized by pain and sensorimotor deficits, underscore the considerable challenges faced by patients with chronic pain when attempting to re-enter the workforce (Córdoba-Mosqueda et al., 2023). Our data show that a substantial amount of patients were able to return to work after surgery.

A major strength of our study is the selection of patients based on a a recently reached consensus for classification of nTOS and usage of a standardized questionnaire allowing for structural comparisons with the general population as well as for cross-country comparisons. However, the generalizability of our results may be limited due to the retrospective single-center design as well as the relatively limited patient number. Also, a longer follow-up interval could have been able to give a broader picture in terms of potential recurrence risks.

## 5 Conclusion

Our study shows that a substantial amount of nTOS patients profited from microsurgical decompressive surgery via the supraclavicular approach without routine first rib resection. This benefit was present not only for pain and neurological function but had substantial impact on health-related QoL as well as on self-care, ability to perform usual activities, pain, and anxiety. After nTOS surgery via the supraclavicular approach without routine first rib resection, QoL levels comparable to those of the general population were reached. Whether the careful selection process according to recent consensus statements contributes to the favourable outcome in our series remains speculative at this point.

## Data availability

Data is available on reasonable request.

## Disclosure of funding

N.F.D. was funded by the institutional Rahel Hirsch and Lydia Rabinowitsch scholarships, and received public body funding for the project GoSafe (Horizon, 2020). No funding bodies had any role in study design, data collection and analysis, decision to publish, or preparation of the manuscript.

## Declaration of competing interest

The authors declare that they have no known competing financial interests or personal relationships that could have appeared to influence the work reported in this paper.
